# Examining Brain Networks in Prescription Opioid Users

**DOI:** 10.4172/2329-6488.1000e137

**Published:** 2017-04-08

**Authors:** Suchismita Ray

**Affiliations:** Center of Alcohol Studies, Rutgers, The State University of New Jersey, NJ, USA

## Introduction

Prescription opioid dependence has reached epidemic proportions in the U.S. and internationally [1]. Overdose deaths involving prescription opioids have increased significantly since 1999; indeed from 1999 to 2015, more than 183,000 people have died in the U.S. from overdoses related to prescription opioids [2,3]. It is well known that drug addiction is a disease of the brain [4] and thus, there is a critical need to get a fundamental understanding of the effects of chronic prescription opioid use on the human brain specifically on brain function and structure.

Research has identified brain regions specifically associated with psychological processes underlying craving and substance use. A few studies have investigated cue reactivity [5–7], and attentional bias [8,9] to prescription opioid related picture cues in chronic users. Cue reactivity is defined as an observable, classically conditioned response to alcohol and drugs [10] that correlate modestly with self-reported craving [11], and attention bias refers to giving drug related cues an increased priority in cognitive processing [12]. Bunce et al. compared recently withdrawn Prescription Opioid Dependent (POD) patients with the patients in supervised residential care for 2–3 months using a prescription drug cue reactivity task. They monitored the prefrontal cortex with functional near-infrared spectroscopy during the cue reactivity task. The recently withdrawn patients showed increased activation to pill stimuli in the right dorsolateral prefrontal cortex relative to extended care patients. Also, POD chronic pain patients evidenced a significant attentional bias towards prescription opioid related cues whereas non-dependent users did not show any significant attention bias [8]. These studies suggest that prescription opioid cues in the environment acquire salience, initiate arousal, and bias attention, as do other types of drug related cues.

Yet, critical to our understanding of the neurobiology of chronic prescription opioid use is defining brain connectivity, that is, how the brain regions interact as opposed to focusing on the functionality of individual regions of interest in isolation. There are only two brain connectivity studies that have examined functional connectivity in the brain of the POD patients [8,13]. Resting State Functional Connectivity (RSFC), measured by the correlation of spontaneous fluctuations of Blood Oxygen Level-Dependent (BOLD) signals in different regions of the resting brain, is believed to provide a measure of the brain’s functional organization [14,15]. Individuals with more intense chronic pain showed decreased RSFC between the perigenual anterior cingulate and the default mode (cognitive control) network likely reflecting reduced ability to govern pain-related thought processes [8]. Bilateral structural volumetric loss in the amygdala, significantly decreased anisotropy in axonal pathways specific to the amygdala, and significantly decreased RSFC for seed regions that included the anterior insula, nucleus accumbens and amygdala subdivisions have been demonstrated in POD persons compared to healthy controls [13].

However, the effects of long term prescription opioid use on the large scale brain networks that have implications in drug addiction have not been examined. These large scale brain networks include default mode network [16]; salience network [17]; lateral visual network [17]; dorsal attention network [18]; and the Drug Cue Processing Network (DCPN) which is a part of the mesocorticolimbic and nigrostriatal system implicated in drug seeking behavior and continuation of drug use [19,20]. The DCPN captures elements of the default, salience, and executive control networks. Drug addiction causes changes in specific regions in the mesocorticolimbic and nigrostriatal system and these changes are manifested through clinical features of compulsive drug seeking, relapses despite negative consequences of using a drug, inhibitory control deficit and reward disturbances [19]. One drug cue reactivity study [6] has examined isolated brain area activation in response to prescription drug cues, but no study has examined structural and functional connectivity among brain areas within the DCPN and other large scale brain networks in prescription opioid users. It is essential to conduct such a study as the results will be the key to our understanding about brain networks reorganization due to prescription opioid addiction.

## Directions for Future Research

The literature cited above suggests the following directions for future neurocognitive research on prescription opioid addiction. First, studies would do well to include a multimodal assessment of structural and functional brain changes in prescription opioid users’ brain networks including DCPN. This would be achieved by using functional MRI and structural Diffusion Tensor Imaging (DTI) techniques and structural, functional and effective connectivity (which reveals a causal influence of one brain area on another) analysis to understand changes in brain networks due to long term prescription opioid use. It would also be important for future researchers to collect for the first time the structural, functional and effective connectivity data between the regions within different brain networks in PODs and matched controls. Data collection should be geared toward developing both pharmacologic and behavioral therapies in the treatment of relapse prevention in individuals with prescription opioid use disorder. Finally, translational constructs linking research to clinical applications should be explicated. Specifically, how therapies can be developed from brain connectivity research to change the neuroplasticity within the DCPN and other large brain networks. Restoring normal functioning of these networks holds promise in promoting abstinence from drug use in this high-risk population.
